# People having hematological disorders and hypercoagulability state need extra precautions because of the increased risk of thrombosis after COVID-19 vaccination

**DOI:** 10.3389/fmed.2022.1082611

**Published:** 2023-02-14

**Authors:** Duygu Aydemir, Nuriye Nuray Ulusu

**Affiliations:** ^1^School of Medicine, Koc University, Sariyer, Istanbul, Türkiye; ^2^Koc University Research Center for Translational Medicine (KUTTAM), Sariyer, Istanbul, Türkiye

**Keywords:** hematological disorders, thrombosis, hypercoagulability, COVID-19, vaccination

## Introduction

Millions of people have been infected and died because of the COVID-19 pandemic since December 2019, which is not the first pandemic the world has faced (1). Older people and individuals with comorbidities such as cancer, diabetes mellitus, cardiovascular diseases, hematological disorders, immune disease, enzyme deficiencies, and chronic respiratory diseases are reported to be more vulnerable to COVID-19, making them major risk groups (2–5). According to the literature, people with hematological disorders and hypercoagulability states are more vulnerable to COVID-19 infection because of the increased risk of adverse health effects and mortality. However, the impact of the COVID-19 vaccination has not been widely investigated in patients having hematological disorders such as glucose 6-phosphate dehydrogenase (G6PD) deficiency, thalassemia, sickle cell disease (SCD), pyruvate kinase enzyme deficiency (PKD), thrombophilia, hypereosinophilic syndromes, Glanzmann syndrome, sticky platelet syndrome, immune thrombocytopenia, and antithrombin deficiency (6, 7).

COVID-19 infection and COVID-19 vaccines can trigger endothelial damage, inflammation, platelet activation, cytokine storm, oxidative stress, and altered coagulation, contributing to thrombosis in patients (8). Platelets play a major role in thrombosis and inflammatory and immune processes; thus, they have a frontline effect on COVID-19 pathogenesis. Platelet hyperactivity, platelet aggregation, increased platelet–platelet interaction, increased gene expression, adhesion, and spreading in the platelets have been reported in patients infected with COVID-19. On the other hand, platelet hyperactivation has been found in both severe and non-severe forms of COVID-19 disease (9–11). Endothelial injury, coagulation, and thrombosis are directly associated with the disease severity and increased mortality risk in patients infected with COVID-19 and people with hypercoagulability (12, 13). Despite COVID-19 vaccines, people are still infected and die since the virus mutates and spreads quickly. Although vaccination reduces the mortality rate, the severity of the disease, and hospitalization, several studies reported that some COVID-19 vaccines might cause adverse health effects in people having hematological disorders or hypercoagulability state because of increased risk of thrombosis and hemolysis (14).

Therefore, extra caution and supportive care can be crucial for those patients after COVID-19 vaccination (12–15). The severity of COVID-19 infection and possible post-vaccination complications can be monitored *via* coagulation and thrombosis-inducing factors (15) such as increased levels of factor VIII, fibrinogen, plasmin activator inhibitor-1 (PAI-1), Willebrand factor (VWF), tissue factor expression, thrombin generation, and platelet activation and decreased concentrations of antithrombin, protein C, and thrombomodulin (16). In this context, we discussed the possible impact of the COVID-19 vaccination on people with hematological and hypercoagulability disorders correlated with the increased risk of thrombosis.

## COVID-19 vaccine-induced thrombotic events

**Figure 1:**
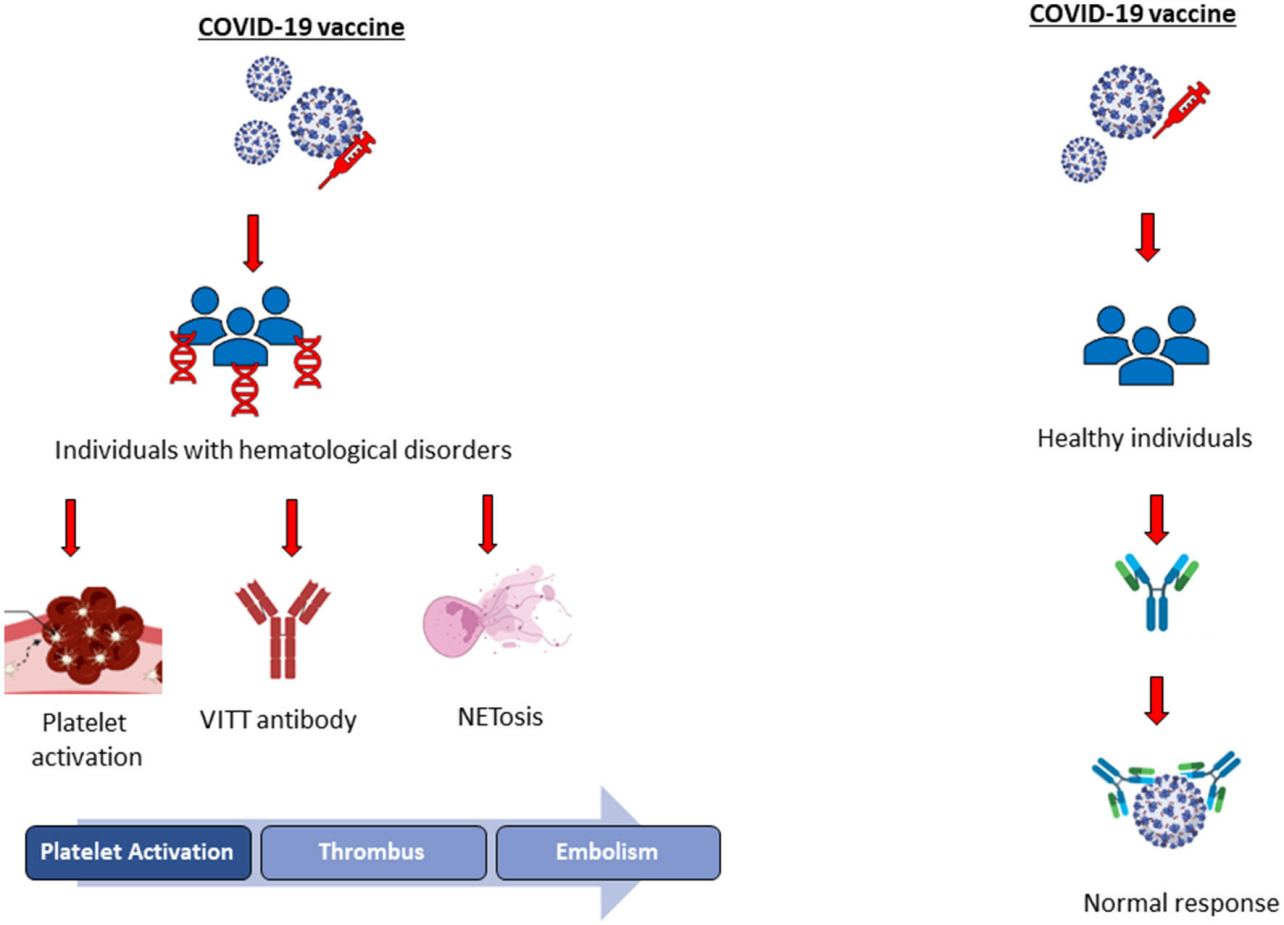
Illustration of COVID-19 vaccine-induced adverse health effects in healthy individuals and people with hematological disorders.

People are vaccinated against COVID-19 infection *via* several types of vaccines, including inactivated, viral vector, and mRNA; however, there is an increasing concern about COVID-19 vaccines about their safety. Thrombosis, platelet aggregation, platelet activity, embolism, and thrombocytopenia have been investigated in healthy individuals vaccinated with adenoviral or mRNA vaccines. Several studies have reported that no vaccine-induced side effects such as persistent platelet aggregation, activation, or plasma thrombin generation have been observed in healthy individuals after COVID-19 vaccination in the long or short term (17–19). On the contrary, viral vector and mRNA COVID-19 vaccines might cause mild-to-severe adverse health effects in people with blood disorders, according to the literature. Thrombosis, portal vein thrombosis (PVT), immune thrombotic thrombocytopenia (ITT), deep vein thrombosis (DVT), vaccine-induced immune thrombocytopenia (VITT), and heparin-induced thrombocytopenia (HIT) are reported as vaccine-induced adverse effects in people with blood disorders; thus, anticoagulation treatment following vaccination (non-heparin anticoagulant and intravenous immunoglobulin) has been recommended by the Expert Hematology Panel in March 2021 and the National Institute for Health Care Excellence (Figure 1) (20). NETosis is reported as the major contributor to VITT and HIT in patients with COVID-19, and NETs formed by neutrophils are involved in the innate immunological response as a first-line defense against pathogens. Increased levels of NETs' formation result in the overactivated immune cells and platelets associated with increased coagulation and endothelial damage, according to the literature (Figure 1) (21). Further studies should be conducted to reveal the mechanisms behind COVID-19 vaccine-induced thrombosis in patients having hematological disorders to prevent vaccine-related complications.

## The impact of COVID-19 vaccines on people with hematological and hypercoagulability state disorders

People with hematological or hypercoagulability disorders such as G6PD deficiency, thalassemia, SCD, pyruvate dehydrogenase deficiency, thrombophilia, and sticky platelet syndrome have a higher risk of developing thrombosis, anemia, hemolysis, and embolism than healthy individuals. G6PD enzyme is the rate-limiting enzyme in the pentose phosphate pathway responsible for producing NADPH involved in maintaining the redox balance in the cell (22–25). Therefore, G6PD enzyme deficiency causes enhanced oxidative stress directly associated with hemoglobin denaturation and intravascular hemolysis (23, 25–27). According to the literature, people with G6PD deficiency can develop life-threatening hemolytic anemia and thrombosis after COVID-19 vaccination or during COVID-19 infection (2–4, 28). On the contrary, PKD is the second most common enzyme deficiency associated with hemolytic anemia, following G6PD deficiency. Since PKD shows clinical heterogeneity due to the type of the mutation, either autosomal recessive or dominant, the disease can be asymptomatic, and hemolysis occurs under stress conditions. Hemolysis, thrombosis, and decreased glycolysis have been found in people with PKD; however, no publication correlates PKD with COVID-19 infection or vaccination. Further studies should be conducted to evaluate the impact of the COVID-19 disease and vaccines on those patients (29, 30).

Thalassemia and SCD are the most common hemoglobin disorders or hemoglobinopathies, and people with hemoglobinopathies require lifelong follow-up and therapy (31). Thrombosis, hypercoagulability state, hemolysis, anemia, and embolism are clinical symptoms of hemoglobinopathies that increase those patients' mortality risk (32). Thalassemia is characterized by the defects of one or more hemoglobin chains and phenotypes of the disease ranging from severe anemia to asymptomatic individuals classified as thalassemia major, intermedia, and minor (33, 34). SCD is another type of hemoglobinopathy characterized by chronic anemia, hemolysis, and vasculopathy (35). Since people with thalassemia or SCD are more vulnerable to COVID-19 infection, they have a higher risk of hospitalization, mortality, and complications (36). Although vaccination enables immunity in both thalassemia and patients with SCD, some people with thalassemia showed vaccine-related adverse effects, including decreased hemoglobin levels and hemolysis (37). Those patients should be followed up closely following the vaccination because of vaso-occlusive crisis (VOC) along with increased levels of white blood cells (WBC) and liver enzyme; in contrast, a significant decrease in hemoglobin and platelet levels has been observed after COVID-19 vaccination (38).

Thrombophilia is characterized by abnormal blood coagulation in almost 50% of people with past thrombotic events. It can be congenital or acquired with clinical symptoms including deep vein thrombosis (DVT) and pulmonary embolism (PE). Protein S deficiency, protein C deficiency, antithrombin deficiency, antiphospholipid syndrome, factor V Leiden (FVL), and hereditary thrombophilia are the types of thrombophilia. Patients with thrombophilia have an increased tendency to clot, venous thrombosis, and thromboembolism because of the hypercoagulability observed in some patients with COVID-19 (39, 40). Antiphospholipid syndrome, also known as Hughes syndrome, is characterized by arterial, venous, or small vessel thrombosis associated with an increased risk of mortality in the patients. It has been reported that antiphospholipid antibodies are found in healthy individuals at approximately 1–5%, which is associated with antiphospholipid syndrome. On the contrary, these antibodies are also determined in patients infected with COVID-19; also Seeley et al. (41) reported that a woman with antiphospholipid syndrome has catastrophic episodes following COVID-19 vaccination (41).

Factor V Leiden is the most common hereditary thrombophilia characterized by a decreased anticoagulant response, increased risk of deep venous thrombosis, and pulmonary embolism (42). People with FVL are more vulnerable to COVID-19 infection since the increased risk of pulmonary embolism and thrombosis; moreover, deep vein thrombosis has been reported after COVID-19 vaccination in a patient with FVL (43). Protein C deficiency is a rare hereditary or acquired life-threatening risk factor that can cause various health problems, such as thrombophilia to venous thromboembolism; it can also be asymptomatic (44). However, there is no publication about protein C deficiency and COVID-19 infection and/or the COVID-19 vaccine in the literature. Protein S deficiency is one of the hypercoagulability syndromes, and the synthesis of this plasma protein depends on vitamin K. Protein S has a central role in protein coagulation, and deficiency of protein S causes thrombotic complications in COVID-19-infected individuals (45, 46). On the contrary, cerebral venous sinus thrombosis has been reported in a patient with protein S deficiency following COVID-19 vaccination; thus, people with thrombophilia should be followed closely after vaccination or during COVID-19 (47).

Immune thrombocytopenia (ITP) is a rare autoimmune disorder with an incidence rate between 1.6 and 3.9 per 100 000 and is commonly found in women. In this disorder, the immune system destroys platelets; therefore, blood cannot clot accurately, leading to the increased risk of venous and arterial thromboembolism. People with ITP are reported to be more vulnerable to COVID-19 vaccination (48–50). Thrombocytopenia and purpuric lesions were observed after several mRNA COVID-19 vaccines, and adverse health effects were reported in patients with ITP (51, 52). Moreover, hyperviscosity syndrome (HVS) is a rare syndrome characterized by high immunoglobins and proteins, which cause an increase in blood thickness. Plasma hyperviscosity has been found in both COVID-19 infection and after vaccination, correlated with disease severity; therefore, people with HVS syndrome can be more vulnerable to COVID-19 infection and vaccination (53, 54).

Sticky platelet syndrome is an autosomal dominant disease characterized by platelet aggregation leading to the increased risk of thrombosis and embolism. According to the literature, people with this syndrome have a higher risk of developing thrombosis during COVID-19 infection (55, 56). Glanzmann thrombasthenia (GT) is a rare autoimmune disorder that can be inherited in an autosomal recessive manner or acquired. Platelet aggregation and decreased coagulation lead to bleeding in patients because of the defects in integrin αIIbβ3. Since thrombosis and embolism are observed in people with GT, they can have severe illness and increased mortality risk during COVID-19 infection, according to the literature (56). However, no detailed study or information about the impact of the COVID-19 infection or vaccination on those patients should be further investigated.

## Conclusion

People are still infected and die because of the COVID-19 virus despite vaccination since the virus mutates and spreads very quickly. Therefore, as major risk groups, older people and individuals with comorbidities should be followed up carefully. People with hematological disorders and hypercoagulability are more vulnerable to COVID-19 infection and vaccination because of the increased risk of thrombosis according to the literature. This is a knife-edge situation because viral vector and mRNA COVID-19 vaccines might cause mild-to-severe adverse health effects but also provide immunity in patients. Therefore, people with hematological disorders are required close follow-up during COVID-19 infection and after COVID-19 vaccines.

## Author contributions

All authors listed have made a substantial, direct, and intellectual contribution to the work and approved it for publication.
